# Whole-genome identification of *HSF* family genes in *Cerasus humilis* and expression analysis under high-temperature stress

**DOI:** 10.3389/fpls.2025.1553187

**Published:** 2025-04-28

**Authors:** Xiaopeng Mu, Jiating Zhang, Chenyi Wang, Liming Chen, Jianying Zhang, Pengfei Wang, Jiancheng Zhang, Bin Zhang, Junjie Du

**Affiliations:** College of Horticulture, Shanxi Agricultural University, Jinzhong, Shanxi, China

**Keywords:** *Cerasus humilis*, *HSF*, phylogenetic analysis, bioinformatics, gene expression

## Abstract

The heat shock factors (*HSFs*) play important roles in activating heat stress responses in plants. *Cerasus humilis* (*Ch*) is a nutrient-rich fruit tree that can resist various abiotic and biotic stressors. However, the *HSFs* in *C. humilis* have not yet been characterized and their roles remain unclear. In this study, 21 *ChHSF* gene members were identified after searching the entire genome of *C. humilis*. Gene structure and motif composition analysis revealed that 16 *ChHSF* genes had only one intron and the motif3 was highly conserved in family of *ChHSFs*. Furthermore, the cis-acting elements analysis indicated that they most *ChHSF*s participate in plant growth and development, abiotic stress responses, and plant hormone regulations. By analyzing the tissue specific transcriptomes, it was found that most *ChHSF* genes had higher expression levels in leaves than in other tissues of *C.humilis*. Notably, the *ChHSF04* gene exhibited a striking 115.5-, 14.4-, and 16.0-fold higher expression in leaves relative to seeds, roots, and fruits, respectively. The high temperature (40 °C) treated *C. humilis* seedlings quantitative real-time polymerase chain reaction (qRT-PCR) was conducted on all *ChHSF* gene members. The results show that the expression of most *ChHSF* genes in the leaves was significantly upregulated and peaked at 12 h under the heat stress and the expression levels of *ChHSF04*, *ChHSF05*, *ChHSF12*, *ChHSF13*, *ChHSF15* and *ChHSF16* exhibited 53-, 33-, 24-, 22-, 43- and 65-fold upregulation, indicating that these genes may play important roles in early response to heat stress in *C. humilis*. These results provide valuable insights into the evolutionary relationship of the *ChHSF* gene family and its role in high temperature stress responses.

## 1 Introduction

Plants are prone to various abiotic stresses, such as unfavorable temperatures, saline-alkali soil, drought, and other adverse environmental conditions (Liu et al., 2023). With the intensification of global climate change, high temperatures have become an important environmental factor restricting the growth and development of woody plants (Zhou et al., 2023). Therefore, plants must alleviate these adverse effects through self-regulation to enable them not only to survive but also to thrive in high-temperature environments (Song et al., 2014; Wan et al., 2019). Thank to evolution, plants have developed a set of mechanisms to respond to high-temperature stress including sensing external temperature changes and reacting quickly to alleviate damage by synthesizing osmotic regulators such as proline and up-regulating the expression of a series of stress response genes (Ahuja et al., 2010; Zanella et al., 2016; Gomez-Pastor et al., 2018; Zhou et al., 2019).

The heat shock transcription factors (HSF), an important transcription factor, was previously reported to participate in high temperature response and other stress responses in plants (Ohama et al., 2016; Wan et al., 2019; Wang et al., 2023). Based on the differences in gene structure, *HSF* genes could be divided into three subfamilies: types A, B, and C (Nover et al., 2001). Type A HSFs were proved to have transcriptional activation functions (Ren et al., 2023). Some studies showed that type B HSFs can assist type A HSFs in performing their functions, whereas few studies were focused on the gene function of type C HSFs (Andrási et al., 2021). In *Arabidopsis thaliana*, overexpression of the *HSFB4* gene can shorten the length of the root, while overexpression of the *HSFA2* gene not only improves the plant’s salt tolerance but also promotes the growth of callus tissue (Begum et al., 2013). In citrus fruits, *CitHsfA7* was the only factor that resulted in a significant lowering of the citric acid content under hot air treatment (Li et al., 2022). In Poplar, stably overexpressing *PeHSFA3* gene exhibited enhanced thermo-tolerance (Ren et al., 2024).

*Cerasus humilis*, a unique dwarfed deciduous fruit tree, belongs to the genus of *Prunus* in Rosaceae, which has a cultivation history of over 3000 years in China (Fu et al., 2021). It has become an important pioneer tree species in arid and semi-arid hilly areas because of its strong tolerance to various abiotic stresses, particularly drought and high temperatures (Wu et al., 2019). However, the heat tolerance mechanisms of *C. humilis* have not yet been studied. Based on the newly released whole genome of *C. humilis*, whole-genome identification of the *HSF* genes family of *C. humilis* was carried out in this study. The gene structure, physicochemical properties, conserved motifs, chromosome location, promoter elements, and phylogenetic relationships of *C. humilis* were analyzed using bioinformatics tools. In addition, qRT-PCR was employed to analyze the expression patterns of all *ChHSF* genes in *C. humilis* plants under high-temperature stress. The results of this study lay an important foundation for the functional identification *ChHSF* genes in the future and subsequent studies of the heat tolerance mechanism of *C. humilis*.

## 2 Materials and methods

### 2.1 High-temperature treatments of *C. humilis*

One-month-old cuttage seedlings of *C. humilis* cultivar ‘Nongda 4’ were taken from the resource nursery of Shanxi Agricultural University and then subjected to room-temperature (25 °C) and high-temperature (40 °C) treatments (Yang et al., 2024). Leaves from treated seedlings for 0 h, 12 h, 24 h, 36 h, and 48 h were collected, frozen in liquid nitrogen, and stored at -80 °C for later use.

### 2.2 Proline content determination

Determination of the proline contents were carried out followed the method described by Abd-ElGawad (Abd-ElGawad et al., 2020). An amount of 0.1 g of liquid nitrogen grinded sample was mixed with 5 mL of 3% sulfosalicylic acid solution, then the mixture was water-bath in boiled water for 15 min. The cooled mixture (1 mL) was transferred to a new test tube and mixed with 2 mL water and 2 mL acetic acid, then water-bathed in boiled water for 1 h to develop color. Subsequently, 5 mL of toluene was added and mixed thoroughly in the dark for 2–3 h. After the layers were completely separated, the absorbance was measured at 520 nm.Proline content per unit of fresh sample (%) = C×Vt/W×Vs×10^6^·100%.

C value is the calculated proline weight from the standard curve (μg/mL), Vt value is the total volume of the extraction solution (mL), Vs value is the sample volume used for determination (mL), W value is the sample mass (g), and the factor 10^6^ is used to convert g into μg.

### 2.3 Genome-wide identification of the *ChHSF* genes

The complete genome sequence of *C. humilis* was downloaded from https://doi.org/10.6084/m9.figshare.11669673. Two methods were used to identify the gene members of the *ChHSF*. First, the HSF protein sequences of *Prunus persica* obtained from the Gramene website (http://www.gramene.org/) were used to conduct local BLAST searches for homologous proteins in *C. humilis*. Secondly, the Hidden Markov Model (PF00447) was downloaded from the Pfam (http://pfam.xfam.org/) website. By running the local hmmsearch software (HMMER 2.3.2) in the local protein database of *C. humilis*, the sequences in that match PF00447 were selected. After removing repetitive sequences, DBDs and coiled-coil structures were studied using CDD (https://www.ncbi.nlm.nih.gov/Structure/cdd/wrpsb.cgi), finally, all HSF proteins with DBDs and coiled-coil structures were retained.

### 2.4 Physicochemical property prediction and phylogenetic analysis of ChHSFs

The number of amino acids, molecular weights, and isoelectric points of the ChHSFs were analyzed using the website ExPASy-ProtParam (https://web.expasy.org/protparam/). Subcellular localization prediction of ChHSFs was performed using the online software PSORT II (https://psort.hgc.jp/form2.html).

Sequences of *Arabidopsis* heat shock factors (AtHSFs) were obtained from the TAIR database (https://www.arabidopsis.org/) and then were aligned ChHSFs using MEGA software (version 7.0). A phylogenetic tree was constructed using the neighbor-joining method (bootstrap = 1000, other parameters set to default); and visualized using EvolView (https://evolgenius.info/evolview-v2/; Subramanian et al., 2019).

### 2.5 Chromosomal localization, gene structure, and conserved motif analysis of *ChHSF* genes

Based on the genome sequence and annotation information of *C. humilis*, TBtools was used to map the *ChHSF* genes onto the chromosomes. Collinearity and gene duplication events in the entire *C. humilis* genome were analyzed using Multiple Collinearity Scan Toolkit (MCScanX) software with default parameters. Gene structure analysis, domain analysis and visualization were performed using TBtools based on data from the CDD website. Conserved motifs in the ChHSFs were analyzed using MEME (https://meme-suite.org/meme/tools/meme).

### 2.6 Promoter analysis of *ChHSF* genes

The 2kb sequences upstream of the transcription start sites of all *ChHSF* genes were extracted and the cis-regulatory elements analyzed the online PlantCARE database (http://bioinformatics.psb.ugent.be/webtools/plantcare/html/).

### 2.7 Analysis of *ChHSF* proteins interaction network

The online website STRING (https://cn.string-db.org/) was used to construct a ChHSF protein interaction networks using *P. persica* as reference (Yang et al., 2023). GO enrichment analysis was performed using eggNOG-mapper (http://eggnog-mapper.embl.de/) and TBTools.

### 2.8 Expression patterns of *ChHSF* in different tissues of *C. humilis*

Transcriptome data for *C. humilis* roots (PRJNA683804), seeds (PRJNA420878), fruits (PRJNA417674), and leaves (PRJNA684437) were downloaded from the NCBI database (https://www.ncbi.nlm.nih.gov/). Tissue specific expression heatmap of *ChHSF* genes were drawn using TBtools based on their FPKM values (Han et al., 2021).

### 2.9 Quantitative real-time polymerase chain reaction

Total RNA from the treated leaves samples was extracted using the CTAB method (Yang et al., 2018). The RNA concentration was determined using a nucleic acid analyzer (Thermo ND2000C) after gel electrophoresis. The cDNA was synthesized using the TaKaRa [PrimeScript™ RT reagent Kit with gDNA Eraser (Perfect Real Time)] reverse transcription kit. QRT-PCR was performed on a 7500 real-time fluorescence quantitative PCR instrument using a SYBR Green (TaKaRa) kit. Each step of the reaction was performed in accordance with the SYBR Green manual. Primers were designed based on the gene sequences (*ChHSF01-ChHSF21*) using Vector NTI (Vector NT1 Advance 11.5). The primer information and gene accession numbers were shown in **Supplementary Tables S1** and **S2**. The internal reference gene was *ChActin* (ouLi_007745). The 20 μL amplification system contained 2 μL cDNA, 0.8 μL of each of the forward and reverse primers, 0.4 μL of ROX Reference Dye II, 10 μL of SYBR, and 6 μL of ddH_2_O. The reaction procedure was as follows: pre-denaturation at 95°C for 30 s; denaturation at 95°C for 15 s, annealing at 58.5°C for 34 s, extension at 95°C for 15 s, 40 cycles, and each treatment was repeated three times. The relative expression of *ChHSF* was calculated with the 2^-ΔΔCT^ method. Data analysis was performed using Excel 2007, Origin 9.0, and SAS 9.2.

## 3 Results

### 3.1 Identification and characterization analysis of *ChHSFs* in *C. humulis*

In total, 21 *HSF* gene members were identified in the whole genome of *C. humilis* and they were sequentially named *ChHSF01* to *ChHSF21* according to their positions on the chromosomes. The molecular weight (MW), theoretical isoelectric point (pI), subcellular localization, and other physicochemical properties were analyzed (**Supplementary Table S2**). As shown in **Supplementary Table S2**, the length of 21 ChHSF proteins varied from 194 aa to 534 aa and their MW ranged from 22.09 kDa to 59.38 kDa, whereas the pI values ranged from 4.67 to 8.53. Subcellular localization analysis indicated that most ChHSF proteins (18 members) were located in the nucleus while the rest (ChHSF04, ChHSF06, and ChHSF19) were located in the cytoplasm, indicating their important roles in the nucleus.

### 3.2 Phylogenetic analysis of *ChHSFs* in *C. humulis*

**Figure 1:**
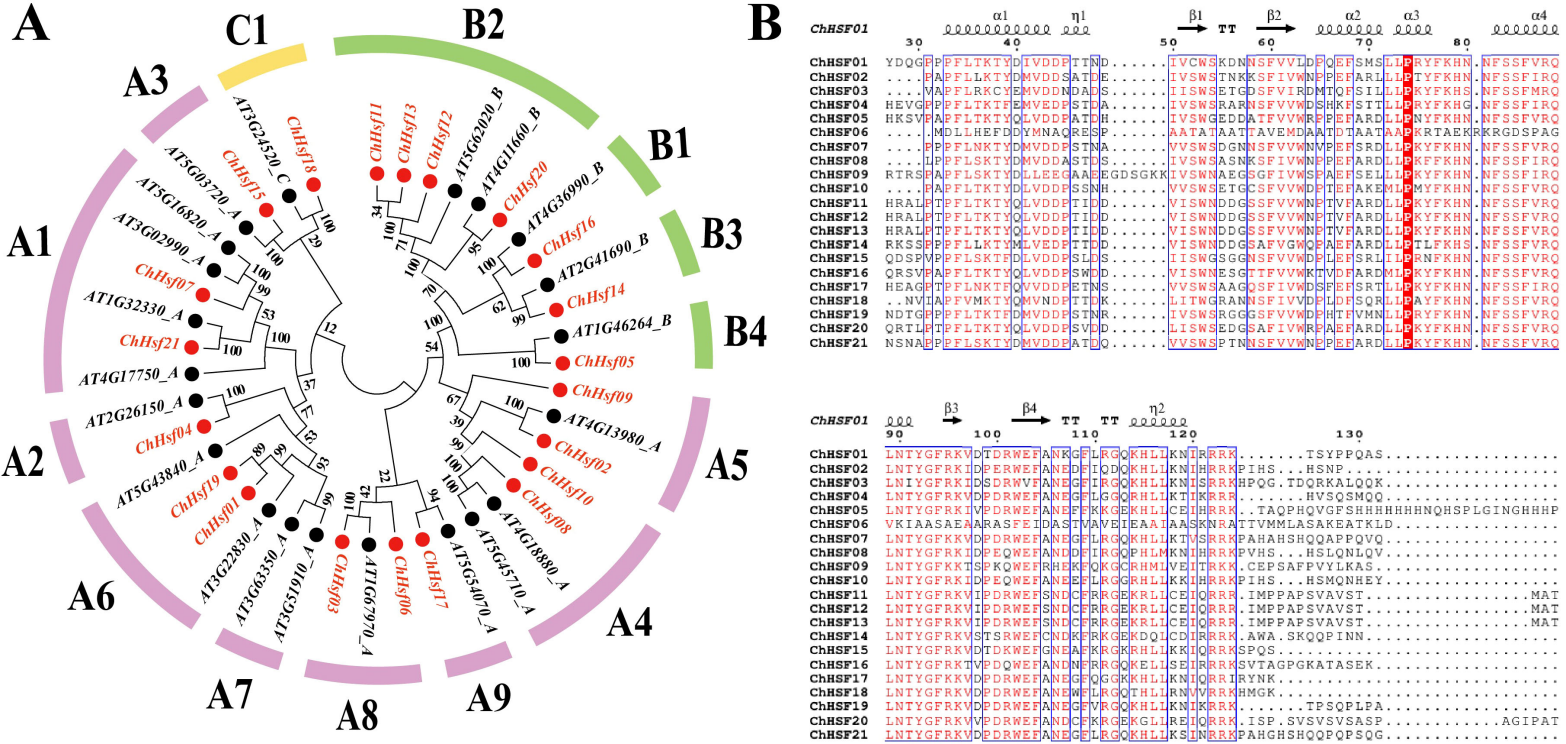
Evolutionary relationship and multiple sequence alignment of the ChHSF proteins. A phylogenetic tree of *C*. *humilis* and *A*. *thaliana* HSF proteins. *C*. *humilis* proteins are denoted in red, whereas *A*. *thaliana* proteins are marked in black **(A)**. Multiple sequence alignment of the DBD domains of the ChHSF protein family **(B)**.

Based on the phylogenetic tree constructed by aligning 21 AtHSFs and 21 ChHSFs, the ChHSFs were divided into three subfamilies (**Figure 1A**). Type A was further divided into 9 subtypes (A1-A9) including 13 family members: *ChHSF01*, *ChHSF02*, *ChHSF03*, *ChHSF04*, *ChHSF06*, *ChHSF07*, *ChHSF08*, *ChHSF09*, *ChHSF10*, *ChHSF15*, *ChHSF17*, *ChHSF19*, and *ChHSF21*. Type B was divided into four subtypes (B1-B4) including seven family members: *ChHSF05*, *ChHSF11*, *ChHSF12*, *ChHSF13*, *ChHSF14*, *ChHSF16*, and *ChHSF20*. Type C included only one family member (*ChHSF18*). The evolutionary tree showed that multiple ChHSFs were tightly clustered with AtHSFs and no ChHSF subfamily members were identified in subtype A7.

The DBD (DNA binding domain) is the most conserved structural domain in *HSFs* comprising three helical bundles (α1, α2, and α3) and four antiparallel β sheets (β1, β2, β3, and β4) (Wang et al., 2024). To further investigate the evolutionary relationship between the structural domains of the ChHSF proteins in the three subfamilies, the amino acid sequences of the 21 ChHSF proteins were subjected to multiple sequence alignment (**Figure 1B**). Most ChHSFs had conserved structural domains and only minor variations were observed in the conserved region except ChHSF06. Interestingly, ChHSF09 has six additional amino acids between α1-β1, which may be related to its specific functional roles and needs to be explored further.

### 3.3 Gene structure and conserved motif analysis of the *ChHSF* family in *C. humilis*

**Figure 2:**
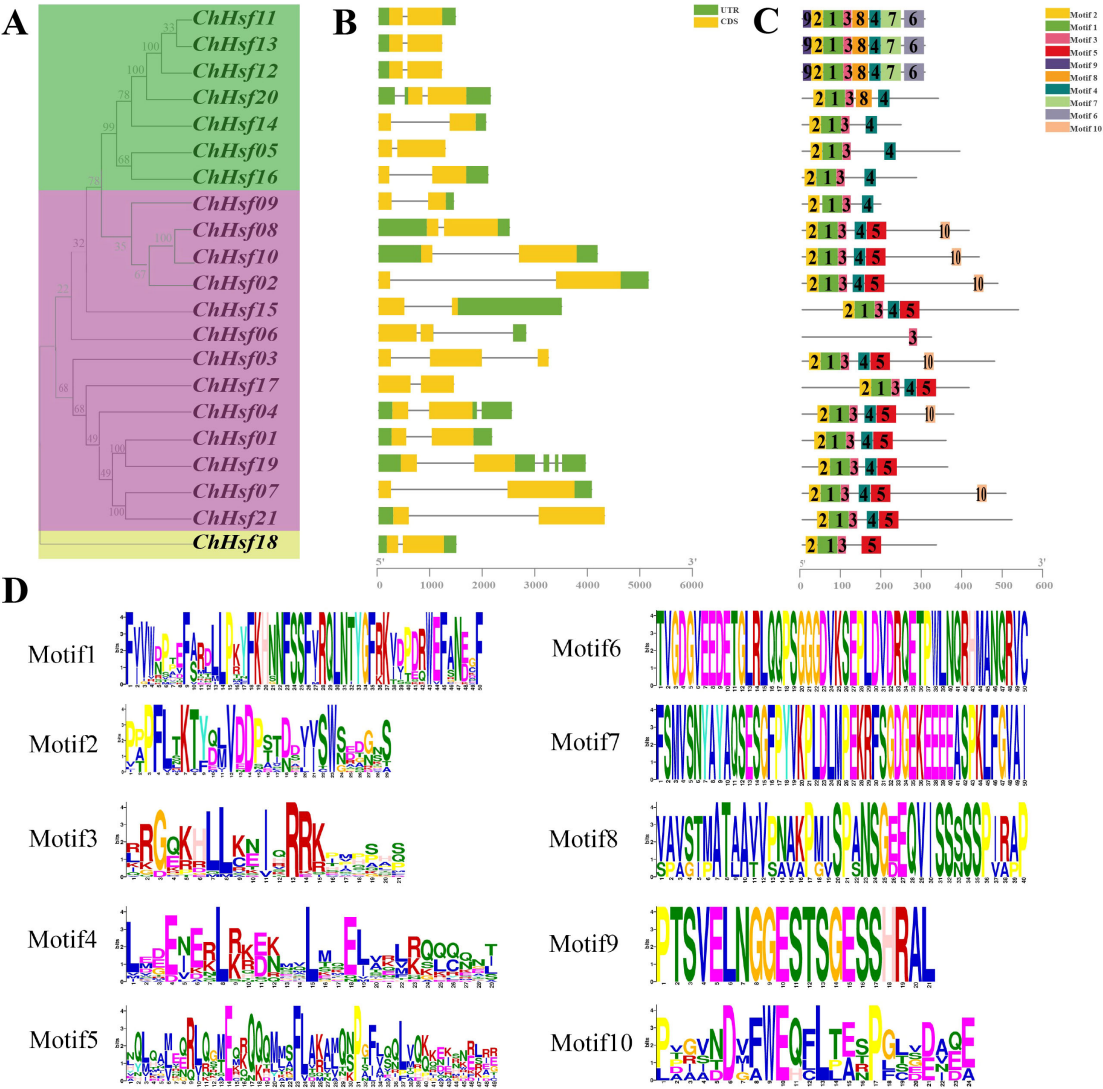
Phylogenetic relationships, gene structure, and protein conservative motif analysis in the *ChHSF* genes family. *ChHSF* Phylogenetic tree **(A)**. *ChHSF* genes structure **(B)**. ChHSF protein conserved motif **(C)**. Conservative motif logo **(D)**.

To understand the structural composition of *ChHSF* genes, a structural map was constructed using their gene sequences (**Figure 2A**). The results indicated that most *ChHSF* genes have only one intron except the *ChHSF03* (2 introns), *ChHSF04* (2 introns), *ChHSF06* (2 introns), *ChHSF20* (2 introns) and *ChHSF19* (4 introns) (**Figure 2B**). Based on the schematic diagram of the conserved motif of the ChHSFs, 10 conserved motifs were identified and the motif 3 was the most conserved motif in *C. humilis* which commonly present in all ChHSFs (**Figures 2C, D**). In group A, ChHSF11, ChHSF12 and ChHSF13 shared the same motif structure (motif 1, 2, 3, 4, 6, 7, 8, 9), while ChHSF05, ChHSF14 and ChHSF16 had the same motif structure (motif 1, 2, 3, 4). The HSF proteins in group B all contained motifs 1 to 4, and ChHSF02, ChHSF04, ChHSF07, ChHSF08, and ChHSF10 shared same motif structure (motif 1, 2, 3, 4, 5, 10). The analysis of conserved motifs revealed a conserved pattern in *HSF* family genes, and within the same group, similar motif compositions were observed.

### 3.4 Chromosome distribution and collinearity analysis of the *ChHSFs* in *C. humilis*

**Figure 3:**
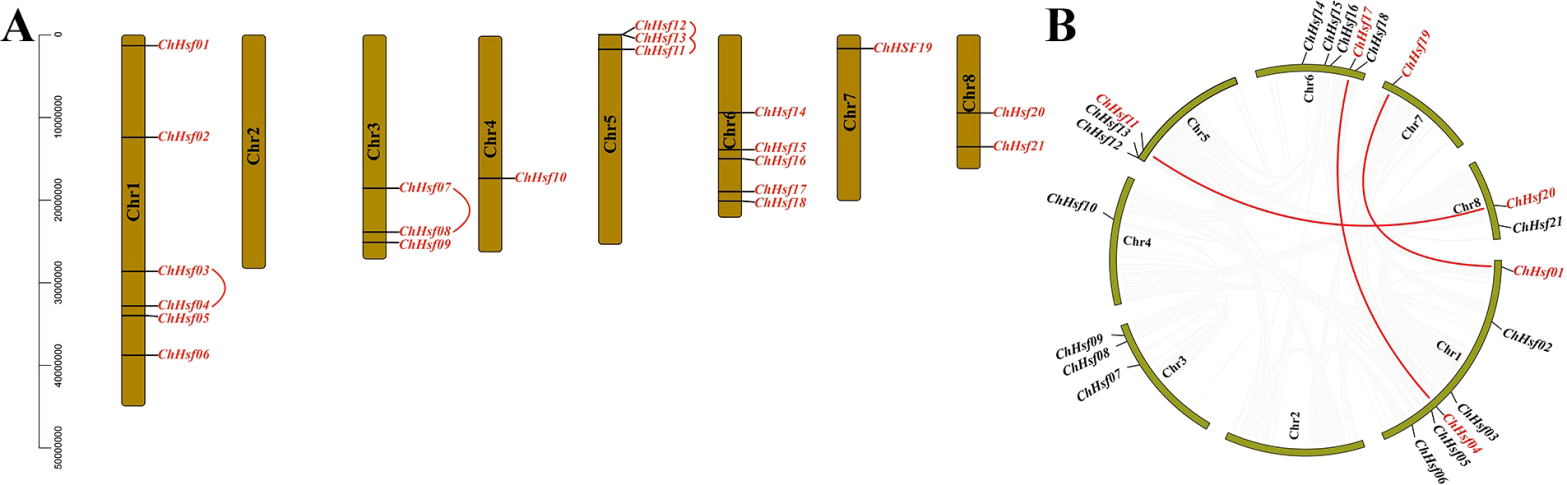
Chromosomal location of *ChHSFs*. Chr 1–8 represents 8 chromosomes respectively; Tandem duplicated of *ChHSFs* were marked with red curves **(A)**; Synteny analysis of *ChHSFs* among chromosomes, red lines represent segmental duplication events **(B)**.

Twenty-one *ChHSF*s were unevenly distributed across the 8 chromosomes of *C. humilis*, and chromosome 1 contained most *ChHSF* gene members (*ChHSF01*-*ChHSF06*), while Chr2 contained 0 *ChHSF* genes. Three tandem duplication events involving seven *ChHSF* genes on chromosome 1 (*ChHSF03/ChHSF04*), chromosome 3 (*ChHSF07/ChHSF08*), and chromosome 5 (*ChHSF11/ChHSF12/ChHSF13*) were observed (**Figure 3A**). Moreover, six homologous sites and three segmental duplication events were identified: *ChHSF01*/*ChHSF19*, *ChHSF04*/*ChHSF17*, and *ChHSF11*/*ChHSF20* (**Figure 3B**). These results suggest that *ChHSF* gene family may have expanded through both tandem and segmental duplication events.

### 3.5 Analysis of *cis*-acting elements in the promoter regions of the *ChHSFs*

**Figure 4:**
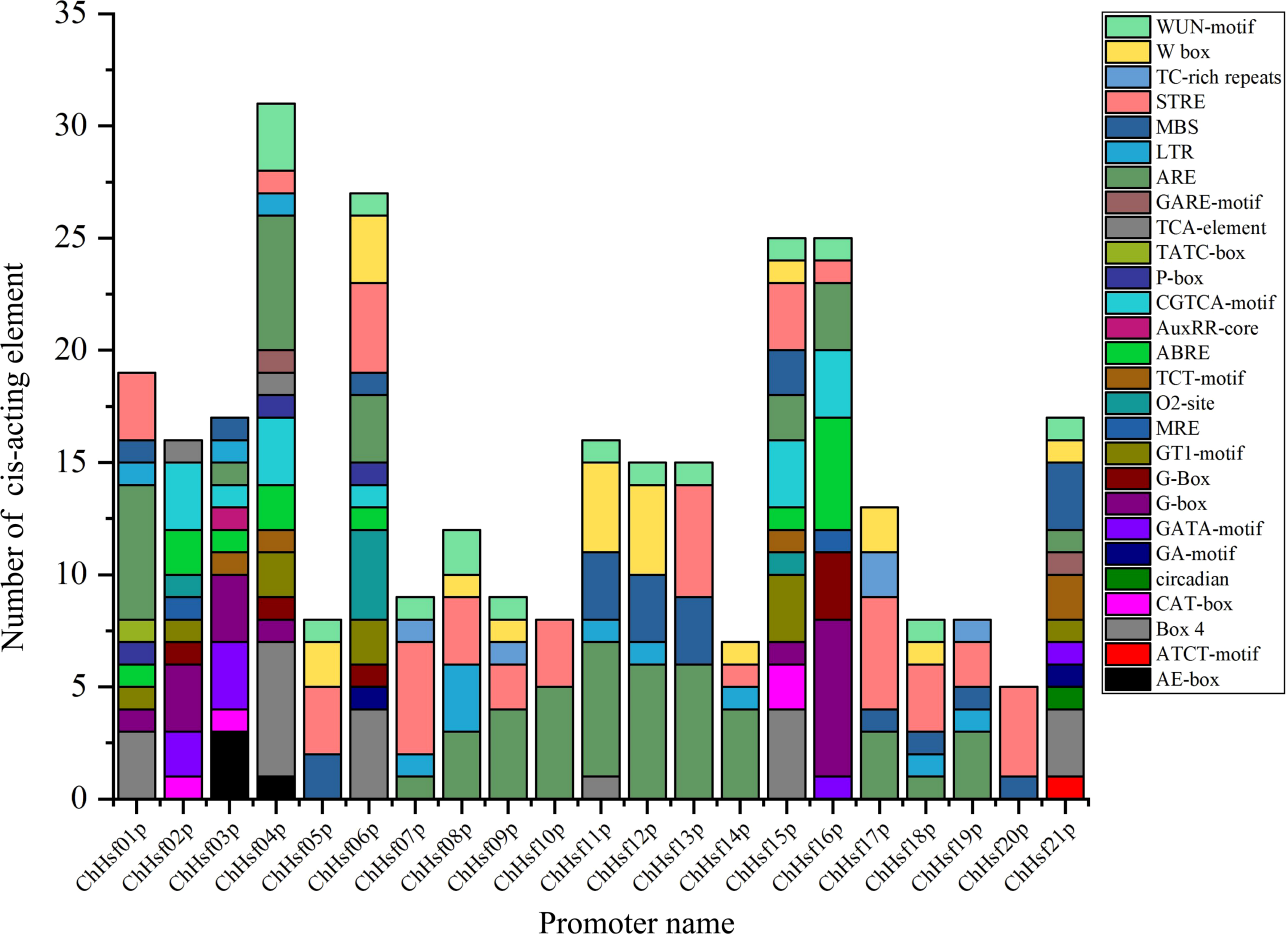
Distribution of cis-acting elements in *ChHSF* gene family promoters.

After predicting the cis-acting elements in the promoter regions of the *ChHSFs* by using the PlantCare online tool, a total of 27 cis-regulatory elements were detected (**Figure 4**), including 13 plant growth and development related elements, 7 hormone responsive elements, and 7 stress responsive elements (STREs). The cis-regulatory element number of *ChHSFs* ranged from 2 (*ChHSF20*) to 15 (*ChHSF04*). Interestingly, most *ChHSF* gene members had STREs in their promoter regions (**Supplementary Table S3**), indicating that *ChHSF*s may play important roles in stress responses in *C. humilis*. However, no heat stress-response cis-regulatory elements (HSE) were detected in the promoter regions of these *ChHSF* genes and it remains unclear whether the expression of the *ChHSF* genes is directly regulated by heat stress.

### 3.6 Protein interaction prediction of the ChHSFs

**Figure 5:**
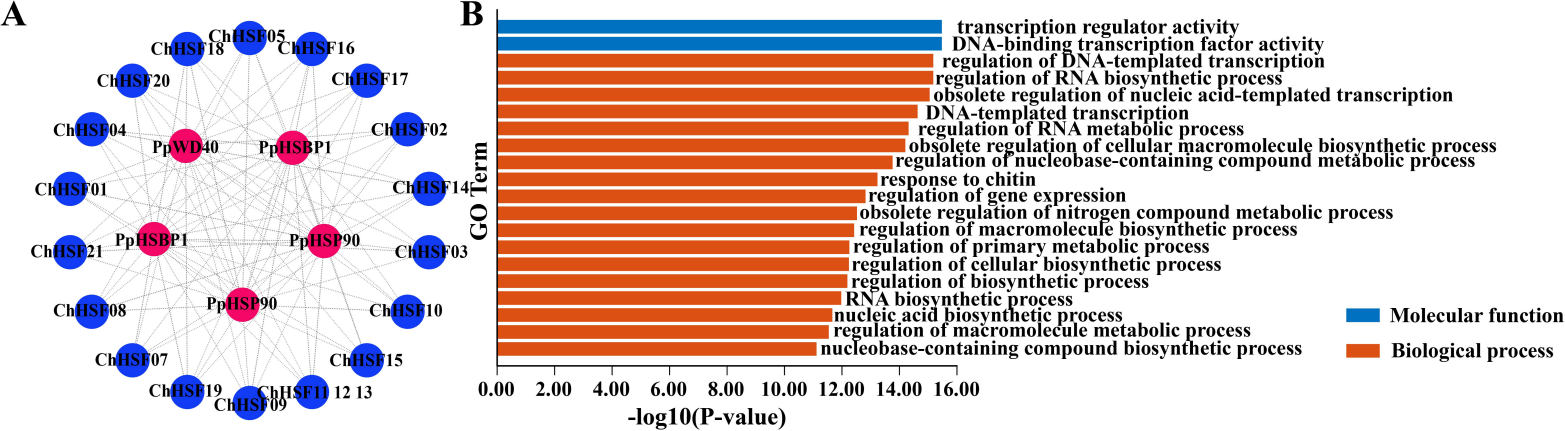
Protein interaction and GO function enrichment analysis of *ChHSF* genes **(A)**. ChHSF protein-protein interaction **(B)**. *ChHSF* gene function analysis, including biological process (BP) and molecular function (MF); biological only displays the top 20 items.

To obtain information on the possible interactions between ChHSF proteins in *C*. *humilis*, an interaction protein network was constructed using the STRING website (**Figure 5A**). The results showed that the three types of proteins had interactions with ChHSF proteins, including heat shock protein (HSP90), HSF-binding protein (HSBP1), and WD repeat protein (WD40). GO function analysis indicated that ChHSFs have the molecular function of transcription regulator activity and DNA-binding transcription factor activity, which are involved in the biological process of the nucleic acid-templated transcription, RNA biosynthetic and metabolic regulations (**Figure 5B**).

### 3.7 Tissue-specific expressions of *ChHSF* genes

**Figure 6:**
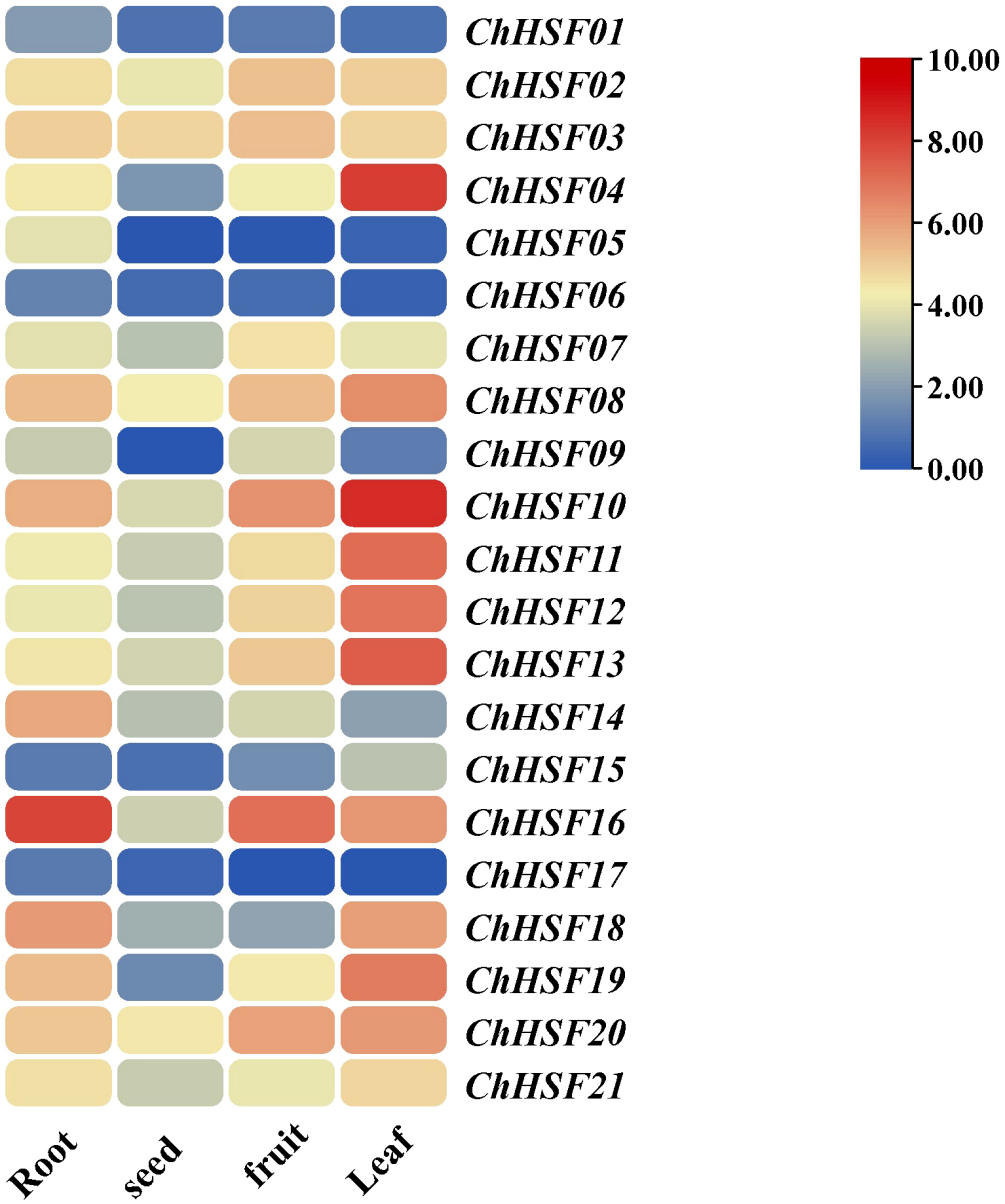
Expression of *ChHSFs* in different tissues of *C. humilis*.

The expression patterns of 21 *ChHSF* genes were analyzed to elucidate their expression characteristics in four different tissues of *C.humilis* (roots, seeds, fruits, and leaves) (**Figure 6**). The results show that most *ChHSF* genes had higher expression levels in leaves than in the roots, fruits and seeds of *C.humilis*. Three *ChHSF* members (*ChHSF01*, *ChHSF06*, and *ChHSF17*) had low relatively low expression levels in all four tissues, indicating that they contribute less than other *ChHSF* genes in regulating the growth and development of these tissues in *C.humilis*. The *ChHSF16* had the highest expression levels in roots and fruits of *C.humilis*, while *ChHSF04* were only highly expressed in leaves. Tissue-specific expression results of the *ChHSF* family genes revealed their functional diversity and complexity.

### 3.8 Growth status and proline content of high temperature treated *C. humilis* cuttings

**Figure 7:**
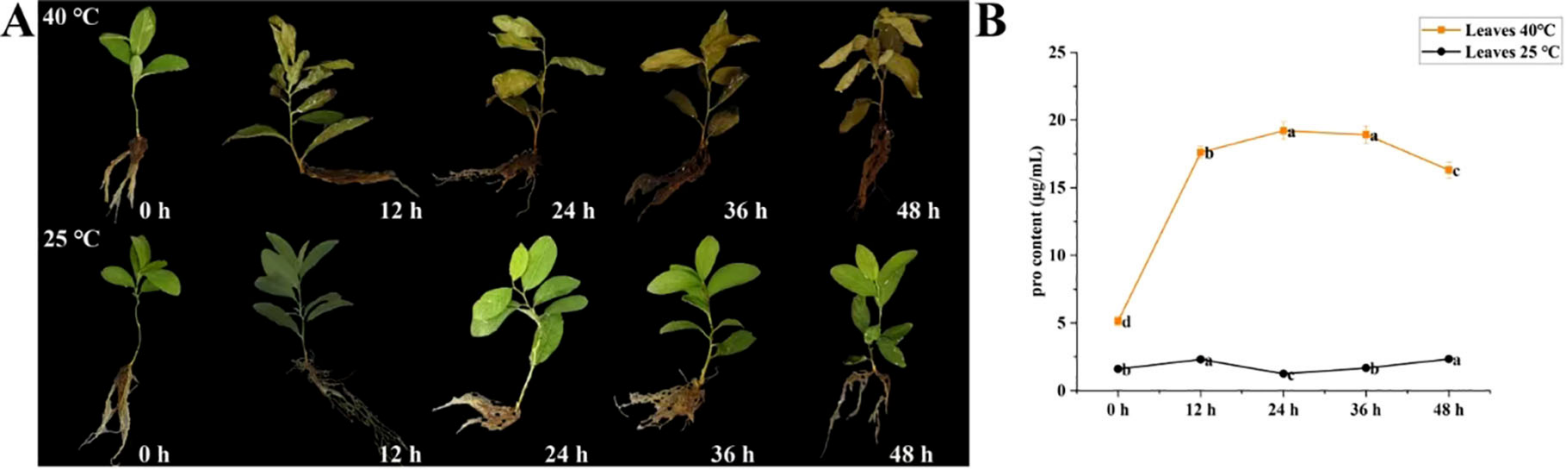
Physiological state of *C*. *humilis* cutting seedlings under high-temperature stress. Growth of *C*. *humilis* cuttings at 25 and 40 °C for 0–48 h **(A)**. Proline contents in leaves of *C*. *humilis* from 0–48 h under high-temperature stress **(B)**.

The growth status of *C. humilis* cuttings under normal temperature (25 °C) and high temperature (40 °C) treatments were documented (**Figure 7A**). The leaves of high temperature treated *C. humilis* cuttings begun to show signs of wilting after 12 h. From 12 to 24 h, the leaves started to turn yellow. After 48 h, the leaves became more fragile and are more likely to fall off. However, the leaves of normal temperature treated *C. humilis* cuttings remained healthy from 0–48 h.

To further verify the effects of high-temperature stress on *C*. *humilis* plants, the proline content in *C*. *humilis* leaves was determined (**Figure 7B**). The proline content in the leaves of *C*. *humilis* under 25 °C treatment kept stable from 0–48 h. However, the proline content in the leaves *C*. *humilis* under 40 °C treatment increased sharply with prolonged stress time, which peaked at 24 h, and then gradually decreases.

### 3.9 Expression analysis of *ChHSF* genes in high temperature treated *C. humilis* cuttings

**Figure 8:**
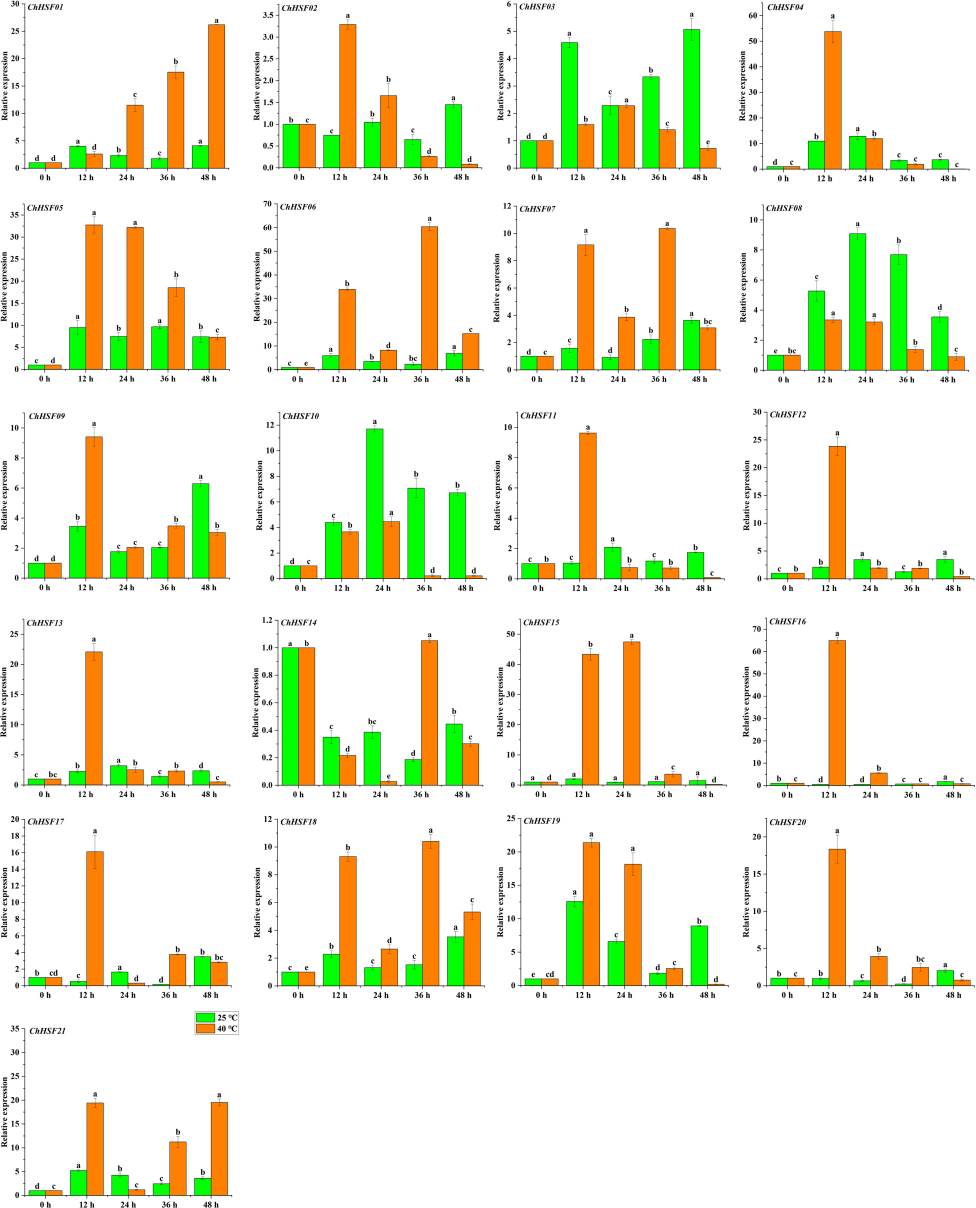
*ChHSF* genes’ expression in leaves of *C. humilis* cutting seedlings under high-temperature stress. Lowercase letters represents significance at p ≤ 0.05.

The expression patterns of 21 *ChHSF* family genes in leaves of normal temperature and high temperature treated *C. humilis* cuttings were determined using qRT-PCR. In the early stage of high temperature treatment (0–12 h), 20 *ChHSF* family genes were up-regulated in leaves of high temperature treated *C. humilis* cuttings, while the *ChHSF14* was significantly down-regulated (**Figure 8**). Noticeably, the expression levels of 10 *ChHSF* family members (*ChHSF04*, *ChHSF05*, *ChHSF06*, *ChHSF12*, *ChHSF13*, *ChHSF15*, *ChHSF16*, *ChHSF17*, *ChHSF19*, *ChHSF20*, and *ChHSF21*) exhibited more than ten-fold upregulation, indicating that these genes may play important roles in early response to heat stress in *C. humilis*. From 12–48 h, most *ChHSF* family members were significantly down-regulated, while the expression levels of *ChHSF15* peaked at 24 h and then decreased in high temperature treated *C. humilis* cuttings.

## 4 Discussion

HSFs are an important group of transcriptional regulatory factor widely present in plants (Wang et al., 2024). To date, *HSF* genes have been identified not only in *A. thaliana* (Nover et al., 2001), but also in many fruit species such as apple (Giorno et al., 2012), strawberry (Hu et al., 2015), Chinese white pear (Qiao et al., 2015), peach (Qiao et al., 2015), grape (Hu et al., 2016), citrus (Lin et al., 2015) and plum (Wan et al., 2019). Moreover, the number of *HSF* family genes varied from 17 (peach) to 35 (carrot) (Huang et al., 2015), the differences in the number of *HSF* family members among different plants may be due to the variations in the retention of *HSF* genes during the evolutionary process of adapting to the environment (Zheng et al., 2024). In this study, 21 *ChHSF* family members were identified in *C. humilis* and they were named as *ChHSF01* to *ChHSF21* according to their positions on the chromosomes. Interestingly, the size of *HSF* family *C.humilis* is bigger than in peach (*Prunus persica*) and plum (*Prunus mume*), indicating that the *C.humilis* is more ancient in evolution.

According to the conserved domains of *Arabidopsis*, the *ChHSFs* family members can be divided into three types: types A, B, and C. Type A includes 13 family members and subfamily A7 does not include *C. humilis* family members, it indicates that these genes are missing during evolution (Ren et al., 2023). Type B includes 7 family members, and type C includes one family member. Analysis of gene structure showed that 21 *ChHSF* genes contained different numbers of introns (1-4). As a part of plant evolution, introns may not only increase the length of genes and the frequency of recombination between genes, but also play an important role in regulation (Shabalina et al., 2010). In contrast, genes without introns have no advantage in the process of species evolution and will delay the regulatory response (Ren et al., 2023), therefore, many *ChHSF* members respond rapidly to stress treatment. Promoter analysis showed that the promoter region of *ChHSF04* had the largest number of cis-regulatory elements among the *ChHSF* family, indicating that *ChHSF04* may be a key gene in the defense against non-biological stress in *C*. *humilis*. Furthermore, no HSE elements were detected in *ChHSFs* promoter regions, suggesting that the expression of *ChHSF* genes related to high temperatures may not be directly induced by heat stress (Tang et al., 2016; Li et al., 2019; Zhang et al., 2020).

Research shows that the expression of the *HSF* gene varies in some species (Song et al., 2014; Liu et al., 2023). In *C. humilis*, several *ChHSFs* display tissue-specific expression patterns. For example, The *ChHSF16* had the highest expression levels in roots of *C.humilis*, while *ChHSF03* and *ChHSF10* were highly expressed in seeds and fruits respectively, suggesting that *HSFs* play widespread roles in the growth and development of different organs and tissues. Studies have shown that HSF gene is involved in high temperature stress response (Fragkostefanakis et al., 2016), in this study, qRT-PCR was used to analyze the expression patterns of 21 *HSF* gene members in leaves of *C.humilis* under high temperature stress, the results showed that 20 *ChHSF* family genes were significantly up-regulated in leaves of high temperature treated *C. humilis* cuttings in the early stage of high temperature treatment (0–12 h). Interestingly, after 12 h of heat stress, the expression of 10 *ChHSF* family genes in leaves was significantly up-regulated in more than ten-fold, indicating that these genes warrant further investigation as promising candidate genes for genetic engineering strategies aimed at enhancing high-temperature resistance in *C. humilis*.

As described in the previous research, changes in proline content can serve as a good indicator of *C.humilis* stress response (Han et al., 2021). By measuring the changes of proline content in leaves at the same time, we found that the proline content in the leaves *C*. *humilis* under 40 °C treatment synchronously increased with expression levels of several *ChHSF* genes, and the functional connections between the proline accumulation and key *ChHSFs’* expressions need to be further studied.

Analysis of the protein interaction network of ChHSF proteins in *C. humilis* shows that three proteins, that is HSP90, HSBP1, and WD40, interact with the HSF protein of *C. humilis*. HSP90 is a major member of HSP and is of great significance as a molecular chaperone (Wei et al., 2024). The HSF regulates the heat shock response of cells, it can bind to HSE under HS, activating the transcription and expression of downstream *HSP* genes (Li et al., 2020). Studies have shown that when plants are exposed to heat stress, the expression of heat shock genes increases, resulting in rapid accumulation of heat shock proteins (HSPs) (Aldubai et al., 2022). The HSF-mediated stress tolerance depends on the regulatory effect of plant HSBPs, which have inhibitory effects on the DNA-binding ability and transactivation activity of *HSF* (Muthusamy et al., 2023). Studies have shown that AtHSBP interacts with AtHSFs to reduce HSF transactivation and HSP gene protein levels (Hsu et al., 2010). In the study of Aldubai et al., it was found that several HSFs, including HsfA2, were expressed in tomato anthers at the early stage of pollen production, indicating that SlHSBP1 may play a regulatory role in HSF development (Aldubai et al., 2022). WD40, also known as the WD-repeat (WDR) protein, is widely present in eukaryotes and plays an important role in abiotic stress and other physiological and biochemical processes (Ke et al., 2023). In summary, the results of this study provide abundant resources for future research on protein interactions between HSF and HSP, WD40, and HSBP1 in *C. humilis*.

## 5 Conclusions

Altogether, we analyzed the gene structures, conserved motifs, cis-acting elements, and expression patterns of the *ChHSF* gene family under high temperature stress. The results provide valuable insights into evolutionary relationships of the *ChHSF* gene family and its role in high-temperature stress responses.

## Data Availability

The original contributions presented in the study are included in the article/**Supplementary Material**. Further inquiries can be directed to the corresponding author.

## Supplementary material

Supplementary Table 1 Primer sequences of quantitative RT-PCR.

Supplementary Table 2 Identification of *HSF* gene family members in *C. humilis*.

Supplementary Table 3 Distribution of the key cis-acting elements in promoters of *ChHSFs.*
