# Supplementary material for: Whole-genome identification of HSF family genes in Cerasus humilis and expression analysis under high-temperature stress
Source: Front Plant Sci. 2025 Apr 28;16:1553187. doi: 10.3389/fpls.2025.1553187 (PMC12066492; doi:10.3389/fpls.2025.1553187)
Supplement: Supplementary Table 1 — Primer sequences of quantitative RT-PCR. [file Table1.docx]

**Table S1.** Primer sequences of quantitative RT-PCR.

| **Gene Name** | **Forward primer（5′–3′）** | **Reverse primer（5′–3′）** |
| --- | --- | --- |
| *ChActin* | CAGTGTCTGGATTGGAGGGT | GCACTTCCTGTCGACAATCG |
| *ChHSF01* | TAAGTTTGTCGTCTTCAGGTGCG | GCTGCCTGACGAAGCTAGAGAAA |
| *ChHSF02* | TGTGAGTGTTTCAGCATCCTTGG | TGCTCCCAAGAAGCCACAAATAA |
| *ChHSF03* | GTGGATCGGTGGCTCCTTTTC | GCAAACACCCAACGATCTGAATC |
| *ChHSF04* | CCAATCTGCTGGCTCTGCAA | CGGAATCGGAGATTGAGACCTTT |
| *ChHSF05* | CTAACATGCGGAGGGAGGCA | CGTCCAAGAGCAGCTCAGTGACT |
| *ChHSF06* | TGGACCTGCTCCATGAATTTGAC | CGATTTCAAAGGAGGCTCTAGCA |
| *ChHSF07* | ATGGAAGGAGTCGAAGACGCG | GCTGCCTGACGAAGCTAGAGAAG |
| *ChHSF08* | ATGGACGAAGTTCAAGGTGGCG | TGTATTGAGCTGCCTGATGAAGC |
| *ChHSF09* | TGGCTGCTACAACTTCTTCAGGC | GGCGGATGAAGCTGGAGAAATTA |
| *ChHSF10* | TCAACTAGCTCGAGTGAGGCGC | CTGCTGCGGCGAATGATATG |
| *ChHSF11* | TGAACGGCGGTGAGTCCACA | GCTGCCTGACGAAGCTAGAGAAG |
| *ChHSF12* | ACACCCTCTGATTAGCCATGTGG | GGAGGCTAGCCCGAAGCTTT |
| *ChHSF13* | ACACCCTCTGATTAGCCATGTGG | GGAGGCTAGCCCGAAGCTTT |
| *ChHSF14* | AGGGTTTGCTGGAATATGTGAGG | CCTGCTTGTTGAAACTTTGCGAA |
| *ChHSF15* | GATGTATCGACTTTCAGCTGGCC | TGGGTTTTGATGTCAGTGCTGGT |
| *ChHSF16* | ATGGCGCAAAGGTCCGTTC | CCATAGGTGTTAAGCTGGCGGA |
| *ChHSF17* | TGGGGCTTAAGATCTTGAACTGC | GCCTTCAGTCCAAGTCCTTGAAA |
| *ChHSF18* | GGAGGAGAACAACAACGTCATCG | CATATGTGTTAAGCTGGCGAACG |
| *ChHSF19* | ACCCAGTGAAGGAAGAGTTCCCG | CCAAACAACAAAGCTTCCACCTC |
| *ChHSF20* | GGTTAACGGCGCTGGAGGAA | CGGACGAAGCTGGAGAAGTTGTT |
| *ChHSF21* | ATGGGAGGTGCTAATAACAACGG | CACGAAGCTGTTGTTGGTGGG |

Table 2 **Identification of *HSF* gene family members in *C. humilis*.**

|  | **Gene Name** | **Accessions number** | **Genetic code** | **Chr** | **Number of amino acids/aa** | **molecular weight/kDa** | **pI** | **Subcellular localization** |
| --- | --- | --- | --- | --- | --- | --- | --- | --- |
|  | *ChHSF01* | ouLi_000222 | Ch1G000222 | Chr1 | 355 | 41.09 | 4.84 | nuclear |
|  | *ChHSF02* | ouLi_001520 | Ch1G001520 | Chr1 | 483 | 54.06 | 5.63 | nuclear |
|  | *ChHSF03* | ouLi_003240 | Ch1G003240 | Chr1 | 475 | 53.10 | 5.29 | nuclear |
|  | *ChHSF04* | ouLi_003958 | Ch1G003958 | Chr1 | 374 | 42.01 | 4.82 | cytoplasmic |
|  | *ChHSF05* | ouLi_004172 | Ch1G004172 | Chr1 | 389 | 43.58 | 6.99 | nuclear |
|  | *ChHSF06* | ouLi_004978 | Ch1G004978 | Chr1 | 319 | 35.13 | 5.72 | cytoplasmic |
|  | *ChHSF07* | ouLi_010749 | Ch3G010749 | Chr3 | 503 | 55.98 | 4.81 | nuclear |
|  | *ChHSF08* | ouLi_011394 | Ch3G011394 | Chr3 | 412 | 46.55 | 5.34 | nuclear |
|  | *ChHSF09* | ouLi_011622 | Ch3G011622 | Chr3 | 194 | 22.09 | 8.53 | nuclear |
|  | *ChHSF10* | ouLi_014057 | Ch4G014057 | Chr4 | 437 | 49.87 | 5.18 | nuclear |
|  | *ChHSF11* | ouLi_015515 | Ch5G015515 | Chr5 | 303 | 33.86 | 5.18 | nuclear |
|  | *ChHSF12* | ouLi_026680 | Ch5G026680 | Chr5 | 303 | 33.86 | 5.18 | nuclear |
|  | *ChHSF13* | ouLi_026730 | Ch5G026730 | Chr5 | 303 | 33.86 | 5.18 | nuclear |
|  | *ChHSF14* | ouLi_019068 | Ch6G019068 | Chr6 | 244 | 27.84 | 5.39 | nuclear |
|  | *ChHSF15* | ouLi_019637 | Ch6G019637 | Chr6 | 534 | 59.38 | 4.96 | nuclear |
|  | *ChHSF16* | ouLi_019791 | Ch6G019791 | Chr6 | 282 | 31.49 | 6.09 | nuclear |
|  | *ChHSF17* | ouLi_020496 | Ch6G020496 | Chr6 | 412 | 45.54 | 6.00 | nuclear |
|  | *ChHSF18* | ouLi_020746 | Ch6G020746 | Chr6 | 331 | 37.75 | 5.81 | nuclear |
|  | *ChHSF19* | ouLi_021487 | Ch7G021487 | Chr7 | 359 | 40.88 | 5.57 | cytoplasmic |
|  | *ChHSF20* | ouLi_025281 | Ch8G025281 | Chr8 | 336 | 36.43 | 4.86 | nuclear |
|  | *ChHSF21* | ouLi_025820 | Ch8G025820 | Chr8 | 518 | 56.21 | 4.67 | nuclear |

**Table S3.** Distribution of the key cis-acting elements in promoters of *ChHSFs.*

| **Gene Name** | **ARE** | **LTR** | **MBS** | **STRE** | **TC-rich repeats** | **W box** | **WUN-motif** |
| --- | --- | --- | --- | --- | --- | --- | --- |
| *ChHSF01* | 6 | 1 | 1 | 3 | 0 | 0 | 0 |
| *ChHSF02* | 0 | 0 | 0 | 0 | 0 | 0 | 0 |
| *ChHSF03* | 1 | 1 | 1 | 0 | 0 | 0 | 0 |
| *ChHSF04* | 6 | 1 | 0 | 1 | 0 | 0 | 3 |
| *ChHSF05* | 0 | 0 | 2 | 3 | 0 | 2 | 1 |
| *ChHSF06* | 3 | 0 | 1 | 4 | 0 | 3 | 1 |
| *ChHSF07* | 1 | 1 | 0 | 5 | 1 | 0 | 1 |
| *ChHSF08* | 3 | 3 | 0 | 3 | 0 | 1 | 2 |
| *ChHSF09* | 4 | 0 | 0 | 2 | 1 | 1 | 1 |
| *ChHSF10* | 5 | 0 | 0 | 3 | 0 | 0 | 0 |
| *ChHSF11* | 6 | 1 | 3 | 0 | 0 | 4 | 1 |
| *ChHSF12* | 6 | 1 | 3 | 0 | 0 | 4 | 1 |
| *ChHSF13* | 6 | 0 | 3 | 5 | 0 | 0 | 1 |
| *ChHSF14* | 4 | 1 | 0 | 1 | 0 | 1 | 0 |
| *ChHSF15* | 2 | 0 | 2 | 3 | 0 | 1 | 1 |
| *ChHSF16* | 3 | 0 | 0 | 1 | 0 | 0 | 1 |
| *ChHSF17* | 3 | 0 | 1 | 5 | 2 | 2 | 0 |
| *ChHSF18* | 1 | 1 | 1 | 3 | 0 | 1 | 1 |
| *ChHSF19* | 3 | 1 | 1 | 2 | 1 | 0 | 0 |
| *ChHSF20* | 0 | 0 | 1 | 4 | 0 | 0 | 0 |
| *ChHSF21* | 1 | 0 | 3 | 0 | 0 | 1 | 1 |
| Total | 64 | 12 | 23 | 48 | 5 | 21 | 16 |
